# *KIF18B* Modulates SKP2 Ubiquitination to Promote Aerobic Glycolysis and Osteosarcoma Progression

**DOI:** 10.3390/ijms27073235

**Published:** 2026-04-02

**Authors:** Haonan Liu, Xin Guo, Chaoxiang Lu, Daifeng Lu

**Affiliations:** School of Clinical Medicine, Harbin Medical University, 157 Health Care Road, Nangang District, Harbin 150023, China; 2023022024@hrbmu.edu.cn (H.L.); 2024022008@hrbmu.edu.cn (X.G.); 2025021847@hrbmu.edu.cn (C.L.)

**Keywords:** *KIF18B*, Skp2, osteosarcoma, ubiquitination, glycolysis

## Abstract

Osteosarcoma (OS) is an aggressive bone malignancy with poor prognosis, characterized by high metastasis rates. Kinesin family member 18B (*KIF18B*), a key protein in cell division and mitosis, has emerged as a potential diagnostic and therapeutic target in various cancers, including OS. This study investigates the role of *KIF18B* in OS progression and its underlying mechanisms. We found that *KIF18B* expression is significantly upregulated in OS tissues and correlates with lymph node metastasis (N-stage) and clinical stage. Knockdown of *KIF18B* inhibited OS cell migration, invasion, proliferation, and tumorigenesis. Mechanistically, *KIF18B* promotes OS survival through the ubiquitin–proteasome system (UPS) by regulating Skp2 protein degradation. *KIF18B* knockdown accelerated Skp2 ubiquitination, leading to reduced Skp2 levels and inhibited OS cell viability and glycolytic metabolism. Overexpression of *KIF18B* enhanced OS cell viability and glycolysis in an Skp2-dependent manner. These findings suggest that the *KIF18B*-Skp2 axis plays a critical role in the metabolic reprogramming of OS cells and serves as a novel prognostic biomarker and therapeutic target in OS.

## 1. Introduction

OS is the most common primary malignancy of the bone [1]. OS is a highly aggressive tumor, known for its rapid proliferation, high malignancy, and a strong tendency to metastasize, particularly to organs such as the lungs [2]. OS is associated with significant disability and mortality rates, often leading to poor patient prognosis [3,4]. Over the past few decades, treatment strategies for OS patients have remained largely unchanged, relying on a combination of surgery and neoadjuvant chemotherapy [5]. While current therapies achieve a 5-year survival rate of up to 70% for localized cases, the overall survival rate has stagnated, with metastasis and recurrence rates remaining high. The 5-year survival rate for recurrent or metastatic cases is only 18.9–20% [6,7,8]. Additionally, the atypical early symptoms of OS, coupled with its metastatic and drug-resistant tendencies, underscore the need for further research into early diagnosis, targeted therapies, and prognostic evaluation [9]. Therefore, there is a pressing need to investigate the molecular targets and associated signaling pathways that regulate OS in order to identify novel therapeutic targets for its treatment.

Kinesins constitute a large superfamily of molecular motors known as Kinesin Superfamily Proteins (KIFs). According to the standard kinesin nomenclature, 45 KIFs have been identified and classified into 14 families (kinesin-1 to kinesin-14) [10,11]. KIFs have been demonstrated to transport organelles, protein complexes, and mRNAs to specific destinations in a microtubule- and ATP-dependent manner [12,13,14]. Furthermore, KIFs participate in chromosome and spindle movements [15,16,17]. Numerous KIFs exhibit aberrant overexpression in various cancer cells. Mitotic Centromere-Associated Kinesin (MCAK), a member of the kinesin-13 family, is upregulated and detectable in breast cancer, colorectal cancer, and glioma tissues [18,19,20]. KIF5B is overexpressed in multiple cancer tissues, including bladder, gastric, skin, and breast cancers [21,22,23,24]. Additionally, KIF14 plays a significant role in the malignancy of various solid tumors, including retinoblastoma and pancreatic cancer [25,26]. Consequently, targeting the kinesin superfamily as diagnostic and prognostic factors represents a promising anticancer strategy. Studies have demonstrated that *KIF18B* is overexpressed in OS tissues. *KIF18B* exerts its function by regulating β-catenin at both transcriptional and post-transcriptional levels [27]; however, the specific mechanisms underlying *KIF18B*’s role in the pathogenesis of OS require further elucidation.

S-phase kinase-associated protein 2 (Skp2, also known as p45) was first cloned from human fibroblasts by Zhang et al. in 1995 [28,29]. Skp2 serves as a key component of the SKP1–Cullin 1–F-box (SCF) complex, an important class of E3 ubiquitin ligases in which the F-box protein is responsible for substrate recognition [30,31]. Recent studies have gradually unveiled the critical role of Skp2 in various cancer types, thereby establishing it as a promising target for therapeutic intervention and drug development [32,33,34,35,36,37,38,39,40,41].

The ubiquitin–proteasome system (UPS) orchestrates substrate degradation and functional modulation of target proteins [42]. Ubiquitin (Ub), a 76-amino acid polypeptide, undergoes enzymatic activation within the UPS cascade and is conjugated to substrate proteins. This ubiquitination process critically regulates diverse cellular activities, including proliferation, migration, apoptosis, protein homeostasis, autophagy, DNA repair, and enzymatic activity modulation [43,44,45]. Substantial evidence implicates UPS dysregulation in cancer pathogenesis [46,47].

Tumor cells promote metabolic adaptation to sustain accelerated proliferation through “metabolic reprogramming”, which frequently manifests as enhanced glycolysis [48]. Metabolic reprogramming encompasses a constellation of metabolic alterations occurring in response to diverse stressors, involving pathways such as amino acid metabolism, lipid metabolism, and glucose metabolism, all critically implicated in the pathogenesis of numerous diseases. Accumulating evidence substantiates that glycolysis plays a pivotal role in tumor growth and progression [49,50]. In normal cells, glucose undergoes catabolism through the glycolytic pathway and the TCA cycle to efficiently generate energy. In striking contrast, cancer cells utilize glycolysis—an inefficient yet rapid ATP-producing pathway—to produce substantial amounts of lactate and pyruvate even under aerobic conditions. This distinct phenomenon is termed the “Warburg effect” or “aerobic glycolysis” [51]. Compared to normal cells, tumor cells exhibit an increased dependence on glucose for energy production, thereby accelerating glucose uptake and lactate production [52]. Although the amount of ATP generated via aerobic glycolysis is substantially lower compared to that produced through aerobic respiration, studies indicate that the expression of glucose transporters (GLUTs) is significantly increased in tumor cells. This elevated expression markedly enhances glucose uptake capacity, thereby supplying abundant substrate for ATP production to meet the demands of rapid tumor growth [53]. Concurrently, the substantial lactate production facilitates alterations in intracellular redox balance, promoting cancer cell invasiveness [52,54,55]. Consequently, the Warburg effect underlies carcinogenic growth, tumor progression, and resistance to cancer therapy, constituting a core component of metabolic reprogramming.

Data from the present study demonstrate that both *KIF18B* and Skp2 function as potential oncogenes, promoting OS progression in vitro and in vivo. Further investigation revealed that *KIF18B* promotes OS progression in an Skp2-dependent manner by regulating Skp2 protein degradation via the UPS. The *KIF18B*-Skp2 axis exerts its oncogenic function in OS development by driving metabolic reprogramming, thereby representing a promising novel therapeutic target in OS treatment strategies.

## 2. Results

### 2.1. Expression of KIF18B in OS and Its Association with Clinicopathological Features and Survival Outcomes 

**Figure 1:**
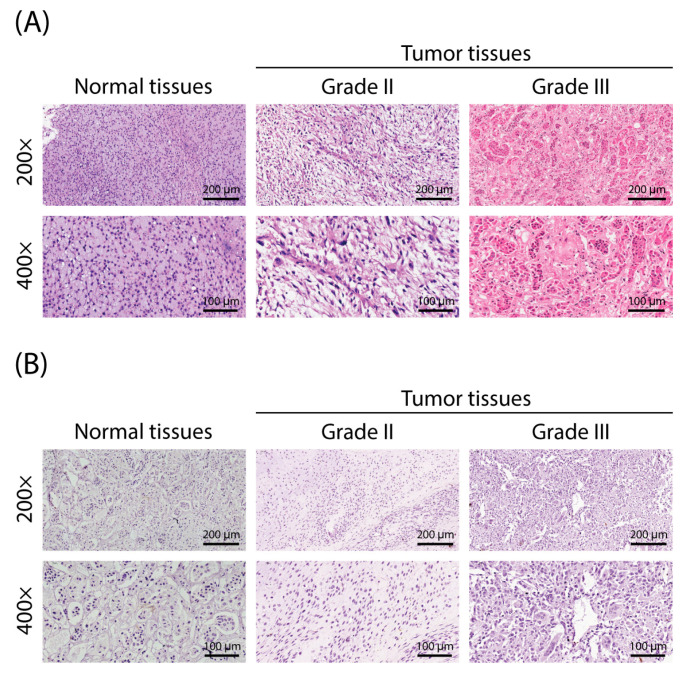
Expression of *KIF18B* in OS and independent validation. (**A**) Hematoxylin–eosin (HE) staining analyses of *KIF18B* in OS and adjacent normal bone tissues to show the general tissue morphology (n = 3; scale bar = 200 μm or 100 μm). (**B**) Immunohistochemical (IHC) staining of *KIF18B* in OS and adjacent normal bone tissues, with DAB as the chromogen to visualize *KIF18B* expression (n = 3; scale bar = 200 μm).

To investigate the role of *KIF18B* in OS, we meticulously prepared tissue microarrays using normal tissue samples (n = 8) and tumor tissue samples (n = 66) collected from clinical patients and performed immunohistochemical (IHC) staining experiments. The staining intensity of *KIF18B* was semi-quantitatively evaluated by two independent pathologists, considering both the percentage of positively stained cells and the staining intensity. Expression was classified as low or high, and any discrepancies were resolved by consensus. By quantifying *KIF18B* expression levels in OS and normal tissues (Table 1) and observing the stained images (Figure 1A,B), we found that *KIF18B* expression in OS tissues was significantly higher than in normal bone tissues (*p* < 0.001) (Table 1). To uncover the potential correlation between *KIF18B* expression and the clinicopathological features of OS, we further conducted the Mann–Whitney U test. The results indicated a significant positive correlation between *KIF18B* expression and both lymph node metastasis (N status) and clinical stage (Table 2). Additionally, through Spearman’s rank correlation analysis (Table 3), we reconfirmed the positive correlation trend between *KIF18B* expression and both the N status and clinical stage, suggesting that as the malignancy of the tumor increases, the expression level of *KIF18B* also rises. Given the potential pivotal role of *KIF18B* in OS, we intend to further investigate its impact on the onset and progression of the disease.

### 2.2. Knockdown of KIF18B Inhibits Progression of OS Cells

**Figure 2:**
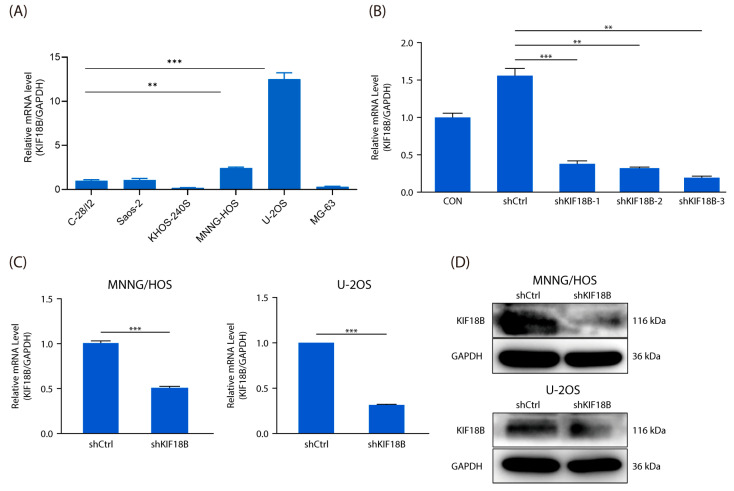

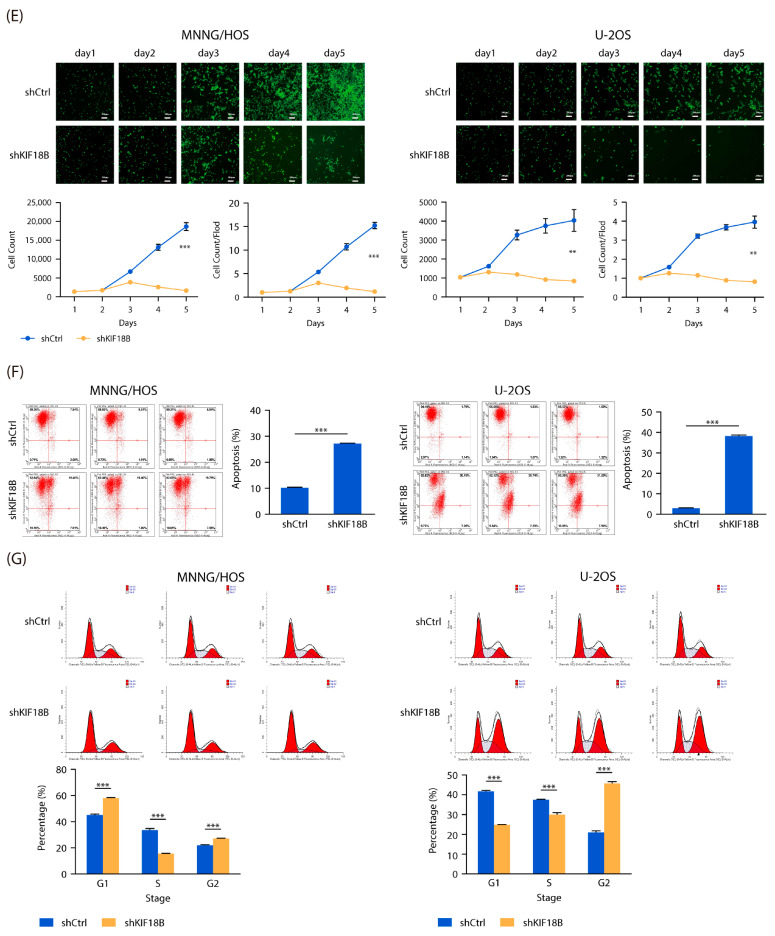

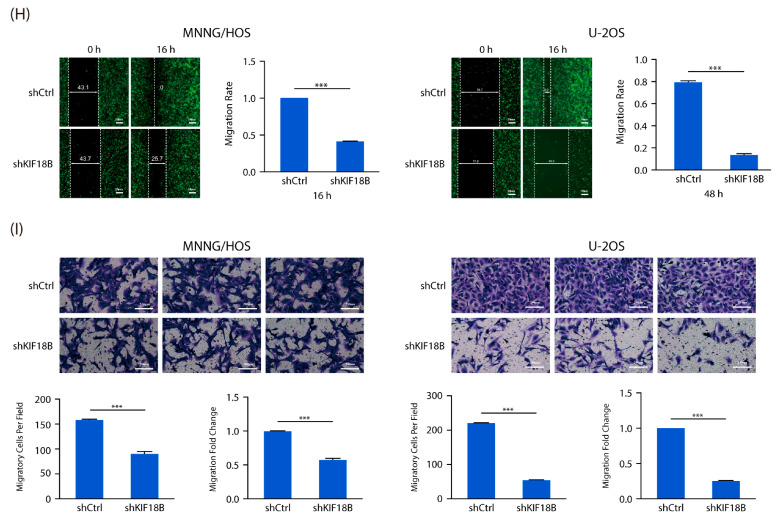
Knockdown of *KIF18B* inhibits the progression of OS cells. (**A**) Detection of background gene expression via qRT-PCR (n = 3). (**B**) Screening for effective interference targets via qRT-PCR (n = 3). (**C**) The knockdown efficiency was detected via qRT-PCR at the mRNA level (n = 3), and (**D**) Western blot on the protein level (n = 3). (**E**) The effect of knockdown of *KIF18B* on MNNG/HOS and U-2OS cell proliferation was examined via Celigo cell counting experiment (n = 3; scale bar = 200 μm). (**F**) Cell apoptosis of MNNG/HOS and U-2OS cells after knockdown of *KIF18B* was analyzed via flow cytometry (n = 3). (**G**) Flow cytometry assays were used to examine the effect of *KIF18B* on the cell cycle. (n = 3). (**H**) Wound healing assays (n = 3; scale bar = 10 μm) and (**I**) Transwell assays (n = 3; scale bar = 200 μm) were conducted to assess cell migration and invasion after knockdown of *KIF18B*, respectively, in MNNG/HOS and U-2OS cells compared with shCtrl group cells. The data were expressed as mean ± SD (n ≥ 3). ** *p* < 0.01 and *** *p* < 0.001.

To gain deeper insights into the impact of *KIF18B* on the biological characteristics of OS, we meticulously planned and conducted a series of pertinent experiments at the cellular level. Panels A and B were performed as preliminary screening experiments to select appropriate cell lines and shRNA constructs for subsequent validation and functional analyses. The results demonstrated that *KIF18B* mRNA expression exhibited variability among OS cell lines relative to normal osteoblasts, with notably elevated expression detected in MNNG/HOS and U-2OS cells, which were therefore selected for subsequent experiments (Figure 2A). Subsequently, based on our screening results (Figure 2B), we selected MNNG/HOS and U-2OS cell lines and successfully established *KIF18B* knockdown cellular models (Figure 2C,D). Further cell counting experiments demonstrated that the knockdown of *KIF18B* effectively suppressed the proliferation of OS cells (Figure 2E). Furthermore, flow cytometric analysis revealed that *KIF18B* knockdown significantly altered cell cycle progression and apoptosis in OS cells. Compared with the shCtrl group, sh*KIF18B*-transfected cells exhibited elevated apoptosis rates (Figure 2F) and G2/M phase accumulation (Figure 2G). The results from both the scratch test and Transwell assay robustly indicated that the knockdown of *KIF18B* significantly impaired the migration (Figure 2H) and invasion capabilities (Figure 2I) of OS cells. In summary, *KIF18B* may play a crucial role in the malignant progression of OS.

### 2.3. KIF18B Targets Skp2 to Promote OS Progression

**Figure 3:**
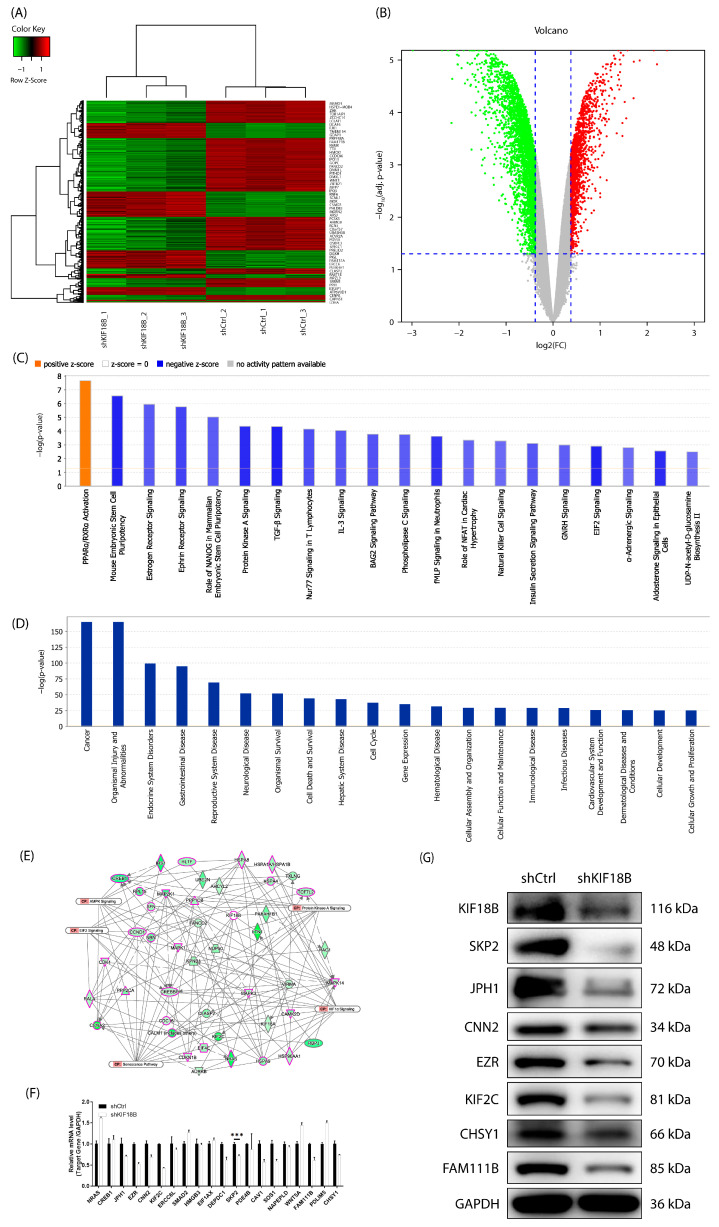

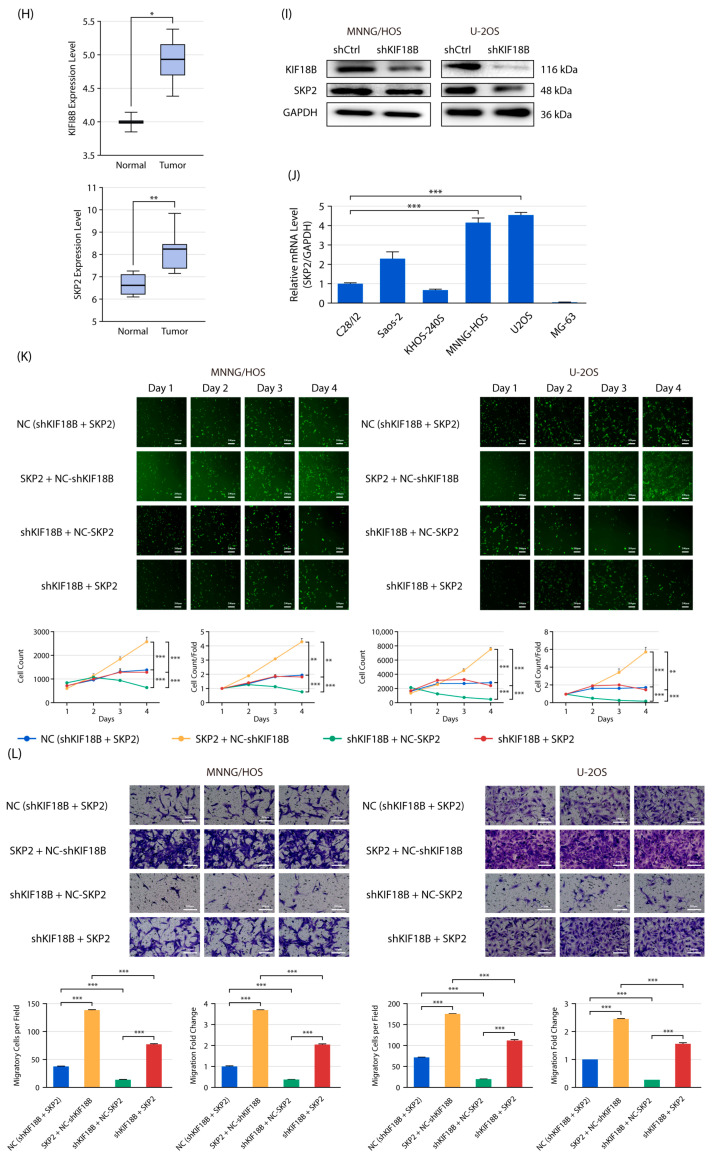
*KIF18B* drives OS cell progression through *SKP2*. (**A**) PrimeView human gene expression array (3 v 3) was performed to identify differentially expressed genes in MNNG/HOS cells. (**B**) A volcano plot illustrates the differentially expressed genes in *KIF18B* knockdown OS cells compared to control cells. The green dots signify downregulated genes (n = 1896), whereas the red dots denote upregulated genes (n = 1053). (**C**) Statistical analysis of classical pathway enrichment revealed significant enrichment of differential genes within classical pathways. (**D**) Statistical analysis of disease and function enrichment demonstrates the significant enrichment of differential genes in specific diseases and functional categories. (**E**) IPA was performed to produce the *KIF18B*-related interaction network. (**F**) Q-PCR and (**G**) Western blot were utilized to detect the expression levels of *SKP2* in OS cell lines (n = 3). (**H**) Independent validation of *KIF18B* and *SKP2* expression using the GEO dataset GSE16091. (**I**) Changes in protein expression after knockdown of *KIF18B* were detected via WB (n = 3). (**J**) Detection of background gene expression via qRT-PCR (n = 3). (**K**) The effect of knockdown of *KIF18B* on MNNG/HOS and U-2OS cell proliferation was examined using the Celigo cell counting experiment (n = 3; scale bar = 200 μm). (**L**) Transwell assays were performed using equal initial cell numbers, and migrated cells were quantified after a fixed incubation time (24 h) (n = 3; scale bar = 200 μm). The data were expressed as mean ± SD (n ≥ 3). * *p* < 0.05, ** *p* < 0.01, and *** *p* < 0.001.

To further explore the regulatory mechanism of *KIF18B* in OS, we performed genome-wide transcriptome profiling using microarray analysis on *KIF18B* knockdown and control OS cells. Based on stringent gene change screening criteria (|fold change| ≥ 1.3 and FDR < 0.05), we identified 1053 upregulated genes and 1896 downregulated genes. These results were visually presented through hierarchical clustering analysis (Figure 3A,B). To systematically elucidate the signaling pathways modulated by *KIF18B*, we performed Ingenuity Pathway Analysis (IPA) on the differentially expressed genes. Beyond the previously identified Protein Kinase A Signaling, the analysis revealed a broad spectrum of significantly altered pathways (Figure 3C). The top 10 most enriched pathways are listed in order of significance, which were predominantly inhibited (negative z-score) upon *KIF18B* knockdown. These included key pathways governing cell pluripotency (e.g., Mouse Embryonic Stem Cell Pluripotency and Role of NANOG), oncogenic signaling (e.g., Estrogen Receptor Signaling and TGF-β Signaling), and immune/cytokine responses (e.g., Nur77 Signaling and IL-3 Signaling). Notably, PPARa/RXRa activation was the sole pathway exhibiting a positive z-score, suggesting a specific metabolic process that was activated following *KIF18B* depletion. This comprehensive pathway profiling indicates that *KIF18B* functions as a central node in a complex network regulating multiple hallmarks of OS pathogenesis. Furthermore, the disease and function enrichment analysis based on IPA revealed that these differentially expressed genes were significantly enriched in cancer-related, organismal injury and abnormalities, and cellular function pathways (Figure 3D). The association between these pathways with *KIF18B* was also established through a network analysis using IPA (Figure 3E). To validate the sequencing results, we selected the top 20 genes with the most significant |fold change| and further confirmed their expression changes in *KIF18B* knockdown and control OS cells through qRT-PCR (Figure 3F) and Western blot (Figure 3G). Several candidate genes showed significant expression changes in qRT-PCR validation; however, *SKP2* was prioritized for further investigation based on its biological relevance and consistency with transcriptomic analysis. To validate our findings, we performed an independent analysis of *KIF18B* and *SKP2* expression using the public Gene Expression Omnibus (GEO) dataset GSE16091. The mRNA expression levels of both *KIF18B* and *SKP2* were significantly upregulated in OS tissues compared to normal bone tissues (Figure 3H). Knockdown of *KIF18B* led to a significant decrease in the expression of the F-box protein Skp2 (Figure 3I). Additionally, our data indicated that Skp2 mRNA expression varied among OS cell lines compared with normal osteoblasts, with relatively higher expression observed in selected cell lines (Figure 3J). This finding replicates and confirms our observations. These findings suggest that Skp2 may be a key target gene regulated by *KIF18B* in OS. To elucidate the potential synergistic effects of *KIF18B* and Skp2 in OS cells, different HOS and U-2OS cell models with *SKP2* overexpression only, *KIF18B* knockdown only, and *SKP2* overexpression with *KIF18B* knockdown were established. The results demonstrated that *SKP2* overexpression significantly enhanced the proliferation capacity (Figure 3K) and migration potential (Figure 3L) of MNNG/HOS and U-2OS OS cell lines. Notably, in these OS cells, *SKP2* overexpression was able to partially reverse the phenotypic changes induced by *KIF18B* knockdown. In conclusion, our experiments provide compelling evidence that *KIF18B* regulates the phenotype of OS cells in an *SKP2*-dependent manner.

### 2.4. KIF18B Facilitates the Tumorigenesis of OS Cells via Skp2

**Figure 4:**
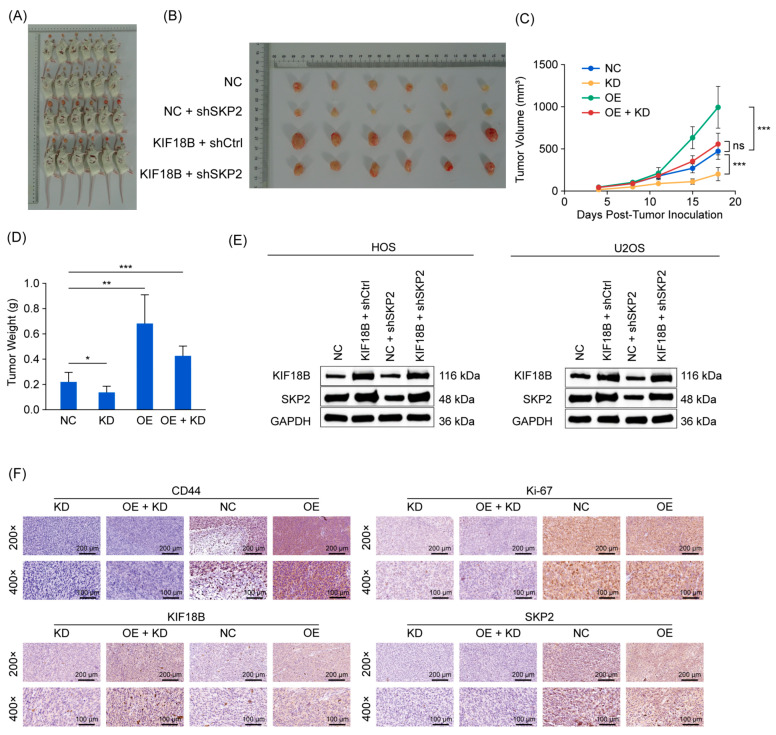
*KIF18B* facilitates the tumorigenesis of OS cells via Skp2. (**A**) Overall view of tumor formation in mice after subcutaneous injection of OS cells (n  =  6 in each group). (**B**) Size, (**C**) volume, and (**D**) weight of the tumors in the KD group (sh*SKP2*), OE group (*KIF18B*), OE + KD group (*KIF18B* + sh*SKP2*), and NC group (unmodified control cells) (n = 6). (**E**) Protein expression levels of *KIF18B* and *SKP2* in each group (n = 3). (**F**) Representative images of IHC staining for CD44, Ki-67, *SKP2*, and *KIF18B* in OS tissue (n = 3; scale bar = 200 μm). The data were expressed as mean ± SD (n ≥ 3). ns, not significant, * *p* < 0.05, ** *p* < 0.01, and *** *p* < 0.001.

To further validate our in vitro findings on the *KIF18B*-Skp2 regulatory axis in OS, we established a xenograft tumor model using MNNG/HOS cells in mice. Specifically, we administered subcutaneous injections to mice with four distinct groups of cells: (1) KD group: cells with *SKP2* knockdown (sh*SKP2*); (2) OE group: cells overexpressing *KIF18B* (*KIF18B*); (3) OE + KD group (*KIF18B* + sh*SKP2*); (4) NC group: unmodified control cells (NC). After euthanasia (Figure 4A) and tumor excision (Figure 4B), the *KIF18B* overexpression group exhibited the largest tumor volume, followed by the *KIF18B* + sh*SKP2* combination group, with the *SKP2* knockdown group showing the smallest tumor volume (Figure 4C). Similarly, tumor weight followed the same trend (Figure 4D). These results suggest that knockdown of *SKP2* can attenuate the tumor-promoting effect of *KIF18B* overexpression. To elucidate the molecular mechanisms underlying these phenotypic alterations, we conducted Western blotting (Figure 4E) and immunohistochemical staining (Figure 4F) on tumor tissues. The results revealed that the expression levels of *KIF18B* and Skp2 were highest in the *KIF18B* overexpression group. Notably, *SKP2* knockdown markedly reduced the expression of *KIF18B*, suggesting that Skp2 plays a pivotal regulatory role in *KIF18B*-mediated tumorigenesis. Collectively, our in vivo findings further corroborate that *SKP2* knockdown attenuates the oncogenic effects of *KIF18B* overexpression in OS cells.

### 2.5. KIF18B Knockdown Accelerated Ubiquitination of Skp2

**Figure 5:**
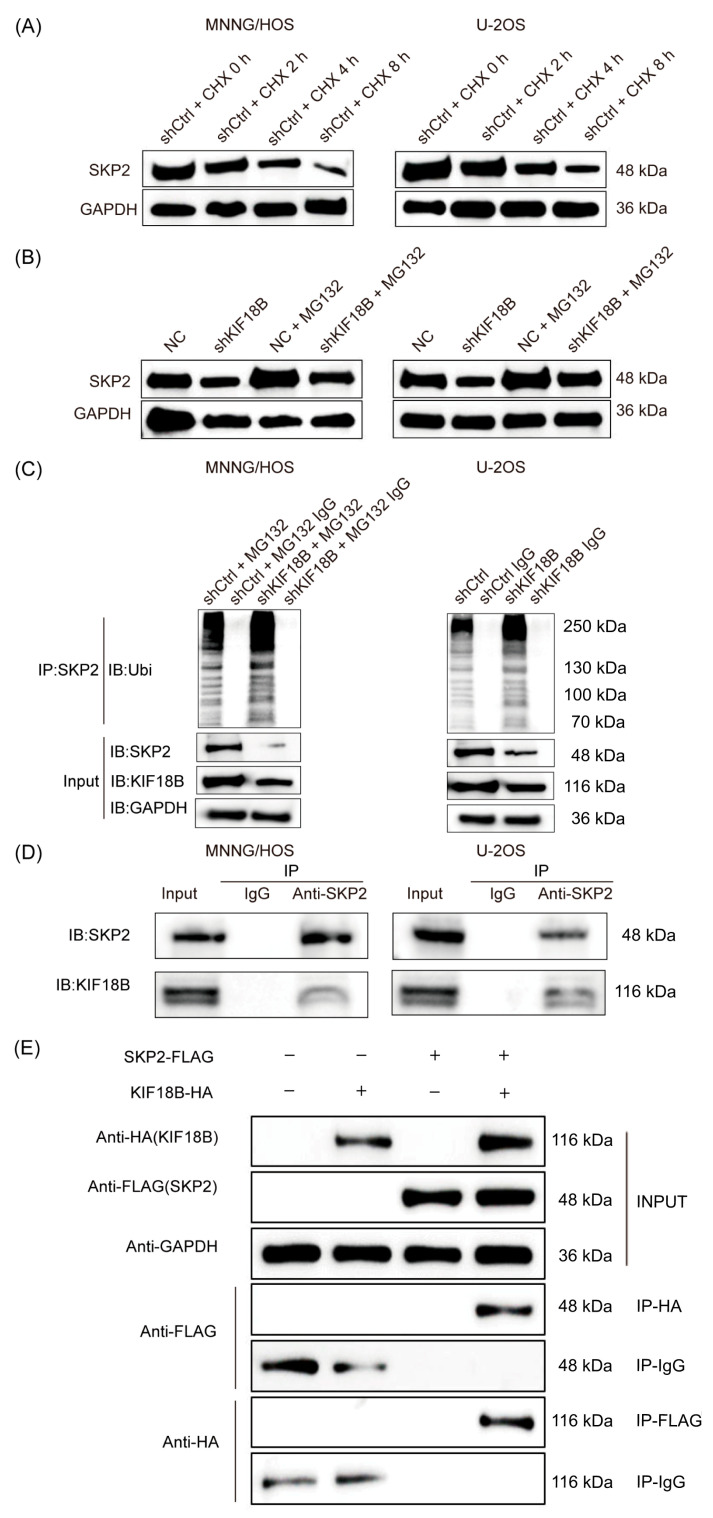
*KIF18B* knockdown accelerated the ubiquitination of Skp2. (**A**) After OS cells in the sh*KIF18B* and shCtrl groups were exposed to the ribosomal inhibitor cycloheximide (CHX) for durations of 0, 2, 4, and 8 h, changes in Skp2 protein expression were detected using Western blotting (n = 3). (**B**) Compared with the sh*KIF18B* group, changes in the expression of Skp2 protein were detected via Western blot in the sh*KIF18B* + MG-132 group after treatment with MG-132, a proteasome inhibitor (n = 3). (**C**) The lysates of MNNG/HOS and U-2OS cells were immunoprecipitated using Skp2 antibodies, and Western blotting was performed to examine the ubiquitination of Skp2 (n = 3). (**D**) Endogenous Co-IP showing the interaction between *KIF18B* and Skp2 in MNNG/HOS cells (n = 3). (**E**) Co-IP using exogenously expressed tagged proteins showing the interaction between *KIF18B* and Skp2 in MNNG/HOS cells (n = 3). (IgG was used as a negative control for immunoprecipitation).

Given that *KIF18B* may regulate the phenotype and tumorigenic effects of OS through Skp2, the molecular mechanism between them has sparked our interest. As a pivotal member of the UPS, the F-box protein Skp2 plays a central role in the progression of various diseases. When OS cells with *KIF18B* knockdown were exposed to the ribosomal inhibitor cycloheximide (CHX), the degradation of Skp2 protein was accelerated (Figure 5A). Intriguingly, upon treatment with the proteasome inhibitor MG-132, the impact of *KIF18B* knockdown on Skp2 protein degradation was attenuated (Figure 5B). These observations indicate that *KIF18B* may regulate the degradation of Skp2 protein via the UPS. Indeed, we observed a marked increase in ubiquitination of Skp2 in OS cells with *KIF18B* knockdown (Figure 5C). Importantly, an interaction between *KIF18B* and Skp2 was demonstrated by co-immunoprecipitation (Co-IP) assays using both endogenous proteins and exogenously expressed tagged proteins (Figure 5D,E). In summary, *KIF18B* knockdown accelerated the ubiquitination of Skp2, leading to downregulation of Skp2 expression.

### 2.6. KIF18B-Skp2 Axis Regulates Aerobic Glycolysis in OS

**Figure 6:**
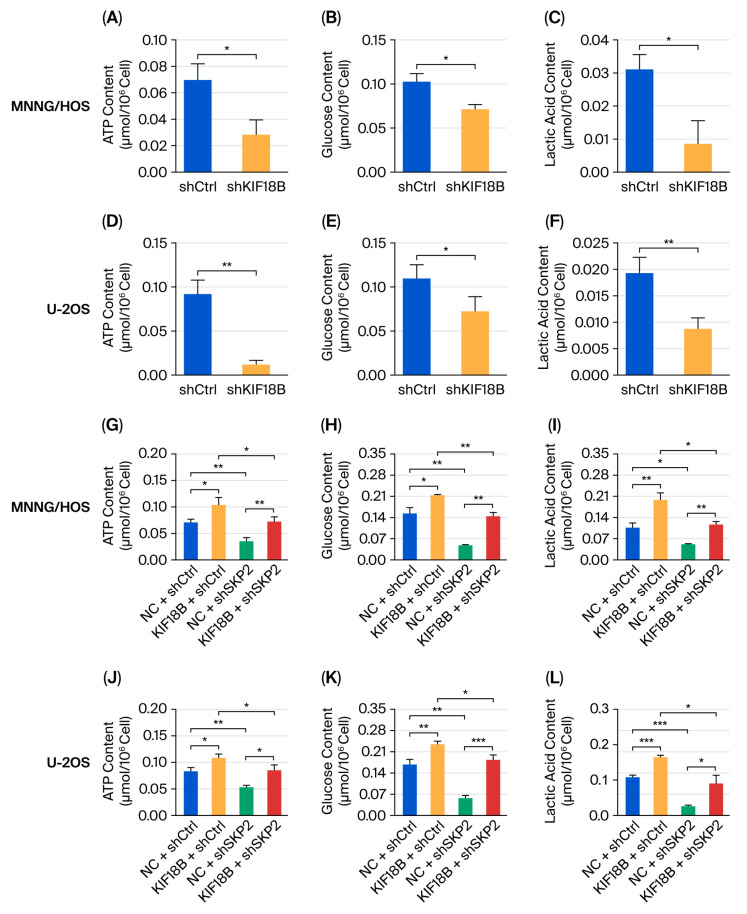

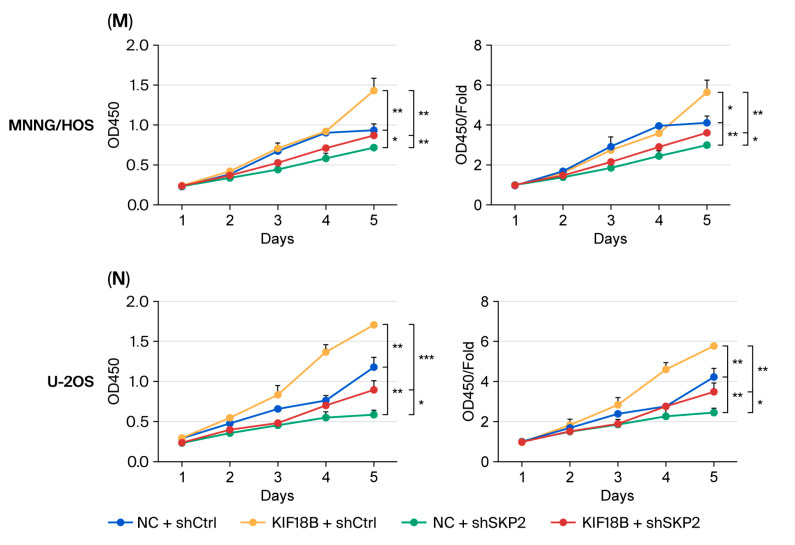
*KIF18B* knockdown accelerated the ubiquitination of Skp2. (**A**–**F**) In MNNG/HOS and U-2OS cells, the levels of ATP, glucose consumption, and lactic acid production were assayed in both the shCtrl group and the sh*KIF18B* group (n = 3). (**G**–**L**) In MNNG/HOS and U-2OS cells, the levels of ATP, glucose consumption, and lactate production were assayed across four groups: NC + shCtrl, *KIF18B* + shCtrl, NC + sh*SKP2*, and *KIF18B* + sh*SKP2* (n = 3). (**M**,**N**) Cell viability was detected via a CCK-8 assay in MNNG/HOS and U-20S cells (n = 3). The data were expressed as mean ± SD (n ≥ 3). * *p* < 0.05, ** *p* < 0.01, and *** *p* < 0.001.

Aerobic glycolysis represents a common characteristic of glucose metabolism in cancer cells. Published studies demonstrate that Skp2 promotes the metabolic shift from TCA cycle utilization toward glycolysis [56]. Given our findings that *KIF18B* deubiquitinates and stabilizes Skp2 protein in OS cells, we next investigated whether *KIF18B* regulates aerobic glycolysis in OS. We first measured glucose consumption, ATP production, and lactate production levels in two experimental groups. Intracellular ATP detection (Figure 6A,D), glucose utilization (Figure 6B,E), and lactate detection (Figure 6C,F) assays showed that *KIF18B* depletion could reduce glycolytic capacity in HOS and U-2OS cells.

To further explore the functional role of the *KIF18B*-Skp2 axis in modulating aerobic glycolysis in OS, we assessed glycolytic capacity (Figure 6G–L) and cellular proliferative capacity (Figure 6M,N) in HOS and U-2OS cells featuring *KIF18B* overexpression and *SKP2* knockdown. Compared with the control group (NC + shCtrl), *KIF18B* overexpression (*KIF18B* + shCtrl) significantly enhanced both glycolytic capacity and cellular proliferative capacity. *SKP2* knockdown (NC + sh*SKP2*) significantly reduced glycolytic capacity and cellular proliferative capacity versus the control group. Crucially, while *KIF18B* overexpression alone enhanced glycolytic capacity and cellular proliferative capacity (*KIF18B* + shCtrl), concomitant *SKP2* knockdown (*KIF18B* + sh*SKP2*) significantly attenuated this pro-glycolytic effect, indicating that *KIF18B*’s glycolytic enhancement largely depends on Skp2. Collectively, these results demonstrate that *KIF18B* overexpression enhances glycolytic capacity and cellular proliferative capacity in OS in an Skp2-dependent manner.

## 3. Discussion

OS represents the most prevalent type of malignant bone tumor worldwide [57]. Currently, the primary treatment for OS involves a combination of surgery and chemotherapeutic agents. Despite advances in therapeutic approaches, the overall survival of OS patients remains poor [6,7,8]. The etiology of OS is largely unknown. Consequently, a deeper understanding of the molecular mechanisms underlying OS development is crucial for identifying novel therapeutic targets and improving patient prognosis.

As a member of the kinesin superfamily, *KIF18B* primarily facilitates microtubule (MT) destabilization. During mitosis, it promotes chromosome congression and restricts microtubule elongation [58]. Structurally, *KIF18B* possesses a characteristic helical neck motif [59]. Conformational switching within this neck region, driven by ATP hydrolysis, enables alternating head domain engagement that propels processive movement along MTs. Cargoes such as membrane-bound vesicles or organelles bind to the kinesin tail domain, facilitating ATP-dependent transport to specific cellular destinations [10]. Notably, this motor protein cooperates with Mitotic Centromere-Associated Kinesin (MCAK) to coordinate microtubule depolymerization [60], while its cargo-transporting activity toward MT plus-ends regulates spindle assembly, chromosome alignment, and MT dynamics [61]. In recent years, the association between *KIF18B* and tumors has attracted significant research attention. Accumulating evidence demonstrates that *KIF18B* is overexpressed in various cancers, including lung adenocarcinoma [62,63], bladder urothelial carcinoma [64], prostate cancer [65], gastric cancer [66], esophageal cancer [67], colorectal cancer [68], hepatocellular carcinoma [69], breast cancer [70], and cervical cancer [71], among others. This overexpression promotes tumor proliferation. While studies have demonstrated *KIF18B* overexpression in OS, a detailed understanding of the regulatory mechanisms through which *KIF18B* governs OS pathogenesis remains lacking.

In our study, *KIF18B* expression was markedly elevated in OS tissues compared with matched paracancerous tissues, demonstrating statistically significant positive correlations with nodal metastasis status (N-category) and advanced clinical staging. Compared with the immortalized human osteoblast cell line C-28/12, *KIF18B* mRNA expression exhibited variability among OS cell lines, with relatively higher expression detected in HOS and U-2OS cells. Independent analysis of the GEO dataset GSE16091 confirmed that the mRNA expression levels of both *KIF18B* and *SKP2* were significantly upregulated in OS tissues compared with normal bone tissues. Based on these findings, we generated stable *KIF18B* knockdown models using shRNA in HOS and U-2OS cells. Functional assays revealed that *KIF18B* depletion induced G2/M phase arrest, promoted apoptosis, and suppressed proliferation, migration, and invasion in both cell lines. Collectively, these results validate that *KIF18B* expression positively correlates with the malignancy of OS, highlighting its potential as a promising therapeutic target for OS treatment. In this study, we further investigated downstream effectors of *KIF18B* and identified and validated Skp2 as a critical mediator of its oncogenic effects in OS. Furthermore, our IPA provides a global perspective on the molecular consequences of *KIF18B* depletion, extending beyond a single pathway. The concerted inhibition of multiple stem cell pluripotency pathways (e.g., Mouse Embryonic Stem Cell Pluripotency and Role of NANOG) suggests that *KIF18B* may be crucial for maintaining an undifferentiated, aggressive state in OS cells, which aligns with the observed reduction in proliferation and migration upon its knockdown. Furthermore, the suppression of established oncogenic drivers such as Estrogen Receptor and TGF-β Signaling offers potential mechanistic explanations for the phenotypic changes, linking *KIF18B* to broader pro-tumorigenic networks. The unique activation of PPARa/RXRa signaling, a pathway involved in lipid metabolism and catabolism, presents an intriguing possibility that *KIF18B* loss might induce a metabolic shift away from anabolic processes that support rapid tumor growth. While Protein Kinase A Signaling remains a key mediator, this multi-faceted pathway analysis posits that the oncogenic role of *KIF18B* is underpinned by its coordinated regulation of a “signaling network” encompassing stemness, survival, and metabolism, rather than a linear pathway.

Skp2, a core component of the F-box protein family, functions as the substrate recognition subunit within the SCF-Skp2 E3 ubiquitin ligase complex. It catalyzes the ubiquitination of key cell cycle regulators—including cyclin-dependent kinase inhibitors (p21 [72], p57 [73]), cyclin E [74], and the proto-oncogene c-Myc [75]—targeting them for proteasomal degradation. Accumulating evidence consistently demonstrates that Skp2 overexpression correlates with adverse outcomes across diverse malignancies: breast cancer [35,76,77], lung cancer [34,36], prostate cancer [37,76], hepatocellular carcinoma [38], colorectal cancer [39], gastric cancer [40,77] and OS [41]. Critically, our data demonstrate that *SKP2* knockdown partially rescues the tumor-promoting effects induced by *KIF18B* overexpression, while *SKP2* overexpression partially reverses the tumor-suppressive phenotype resulting from *KIF18B* depletion in OS. Furthermore, we elucidated the molecular mechanism underlying *KIF18B*-mediated regulation of Skp2. Our experimental data demonstrate that *KIF18B* knockdown promotes Skp2 ubiquitination, consequently downregulating Skp2 protein expression. Thus, we propose that *KIF18B* exerts its oncogenic function in OS by counteracting Skp2 ubiquitin-mediated degradation, thereby stabilizing Skp2 protein levels.

Cancer cells undergo metabolic reprogramming to favor energy production through glycolysis over mitochondrial oxidative phosphorylation, even under aerobic conditions, a phenomenon known as aerobic glycolysis or the Warburg effect [51]. Aerobic glycolysis constitutes a hallmark feature of neoplastic cell bioenergetics [78]. Targeting aerobic glycolysis represents an emerging anticancer strategy, as evidenced by studies demonstrating that shifting cancer metabolism from glycolysis to mitochondrial glucose oxidation effectively suppresses tumor growth [79]. Our results establish that *KIF18B* knockdown significantly attenuates aerobic glycolysis in OS. Critically, *KIF18B*’s capacity to enhance glycolysis is largely dependent on Skp2, with *KIF18B* overexpression amplifying glycolytic metabolism and cellular viability in an Skp2-dependent manner. These findings collectively position the *KIF18B*-Skp2 axis as a central regulator of aerobic glycolysis. Consequently, pharmacological targeting of this axis to reverse cancer-associated aerobic glycolysis may hold therapeutic potential for impeding tumor progression.

Nevertheless, several limitations of this study should be acknowledged. Although metabolic assays indicated enhanced glycolytic activity, we did not directly determine whether glycolysis is required for the functional activity of the *KIF18B*–*SKP2* axis, and future studies using metabolic interventions, such as galactose-based culture conditions, will help clarify this dependency. Although ATP production, glucose consumption, and lactate generation analyses suggested enhanced glycolytic activity, the metabolic characterization in this study was not comprehensive, as our primary aim was to establish the regulatory role of the *KIF18B*–*SKP2* axis rather than fully define metabolic reprogramming. Future studies incorporating Seahorse extracellular flux analysis, isotope tracing, and evaluation of glycolytic enzyme expression will further clarify the metabolic consequences of this regulatory axis. In addition, the dependency of osteosarcoma cells on *KIF18B* was primarily evaluated using shRNA-mediated knockdown, which may involve off-target effects or incomplete silencing; therefore, CRISPR/Cas9-mediated knockout and rescue strategies will further strengthen the genetic evidence. Our functional analyses mainly focused on short-term proliferation assays, and long-term clonogenic or competitive growth assays will be valuable to assess sustained cellular dependency. The effects of *KIF18B* depletion in normal osteoblasts were not examined and should be explored to evaluate tumor selectivity. Mechanistically, while our data demonstrate that *KIF18B* stabilizes *SKP2* through suppression of ubiquitination, the detailed regulatory mechanisms, including the responsible E3 ligase, ubiquitin linkage type, and potential effects on *SKP2* localization or interacting partners, remain to be further elucidated. Given that *KIF18B* functions as a mitotic kinesin, we also cannot completely exclude potential contributions from cell cycle perturbations to *SKP2* destabilization; however, the primary aim of this study was to define the *KIF18B*–*SKP2* interaction and its regulation of ubiquitination-dependent stability, which is supported by our current findings, and future studies incorporating detailed cell cycle analyses will help clarify whether these effects occur independently of mitotic disruption. Finally, although subcutaneous xenograft models supported the in vivo role of *KIF18B*, orthotopic models and metastasis analyses would further enhance translational relevance.

## 4. Materials and Methods

### 4.1. Cell Culture

The human osteoblast cell line C28/I2 and five human OS cell lines (Saos-2, KHOS-240S, MNNG-HOS, U-2OS, and MG-63) were utilized in this study. These cell lines were selected as they represent well-established models for studying OS biology, encompassing a range of genetic backgrounds and phenotypic characteristics. All cell lines were purchased from Shanghai YaoKang Biotechnology Co., Ltd. (Shanghai, China). Upon receipt, cell lines were expanded and cryopreserved at low passages. All experiments were performed with mycoplasma-free cells within 10 passages after resuscitation, and cell identity was confirmed by short tandem repeat (STR) profiling.

All cell lines were maintained in Dulbecco’s Modified Eagle Medium (DMEM) supplemented with 10% fetal bovine serum (FBS; Thermo Fisher Scientific Inc., Waltham, MA, USA) and 1% penicillin/streptomycin (100 U/mL penicillin and 100 µg/mL streptomycin; Thermo Fisher Scientific Inc.). Cells were cultured according to standard protocols. Specifically, based on established cultivation requirements, the medium for HOS and U-2OS cells was further supplemented with 1.0 g/L glucose and 1 mM sodium pyruvate, while the medium for Saos-2 cells was supplemented with 2 mM L-glutamine. The cultures were maintained in a humidified incubator at 37 °C with 5% CO_2_. The culture medium was refreshed every 2–3 days, and cells were subcultured using 0.25% trypsin–EDTA upon reaching 80–90% confluence.

### 4.2. Cell Transfection

Short hairpin RNAs (shRNAs) targeting *KIF18B* and *SKP2* were synthesized and cloned into the lentiviral knockdown vector lv-002 (GenePharma, Shanghai, China). A scrambled non-targeting shRNA was used as the negative control (shNC). The target sequences were as follows:

*shKIF18B-1*: 5′-GAGGAAGAAGCTCCAAGTGTA-3′
*shKIF18B*-2: 5′-CACGTACAACACCCTCAAATA-3′
*shKIF18B*-3: 5′-AAGGGCAAAGACCTGACGTTT-3′
*shSKP2*: 5′-GCAGATGTTGCTGTACAAATT-3′

For overexpression experiments, the full-length coding sequences (CDS) of human *KIF18B* and *SKP2* were cloned into the pcDNA3.1 expression vector (Invitrogen, Carlsbad, CA, USA). Empty pcDNA3.1 vector served as the overexpression negative control (Vector). Cells were transfected using Oligofectamine (Invitrogen Life Technologies, Carlsbad, CA, USA) according to the manufacturer’s instructions. After transfection, stable cell lines were selected using puromycin (2 μg/mL) for 7–10 days. Transfection efficiency was verified by qRT-PCR and Western blot analysis.

Based on different combinations of gene knockdown and overexpression, cells were divided into the following experimental groups:

(1) NC (shNC + Vector)—double negative control group;
(2) *SKP2* + shNC—*SKP2* overexpression alone;
(3) sh*KIF18B* + Vector—*KIF18B* knockdown alone;
(4) sh*KIF18B* + *SKP2*—combined *KIF18B* knockdown and *SKP2* overexpression.

All experiments were performed in triplicate.

### 4.3. RNA Extraction and qRT-PCR

Osteoblasts and OS cells were cultured, and total RNA was extracted using TRIzol reagent (Thermo Fisher Scientific, Waltham, MA, USA). RNA concentration and purity were determined using a NanoDrop 2000 spectrophotometer (Thermo Fisher Scientific). cDNA synthesis was performed using a reverse transcription kit (RR036A; Takara, Japan). qRT-PCR was conducted using SYBR Select Master Mix (4472908; Applied Biosystems, Foster City, CA, USA) in a total reaction volume of 20 μL containing 1000 ng RNA. Amplification was performed on a QuantStudio™ 6 Flex Real-Time PCR System (Applied Biosystems). The primer sequences used were as follows:

*KIF18B* Forward: 5′-CTCCCATGCCATCTTCCAGA-3′
*KIF18B* Reverse: 5′-TGAGCCAGCCAGGTCAATCA-3′
*SKP2* Forward: 5′-ATGCCCCAATCTTGTCCATCT-3′
*SKP2* Reverse: 5′-CACAGTGCTGGTGATGTTCTG-3′
GAPDH Forward: 5′-GGAGCGAGATCCCTCCAAAAT-3′
GAPDH Reverse: 5′-GGCTGTTGTCATACTTCTCATGG-3′

PCR conditions consisted of an initial denaturation at 95 °C for 10 min, followed by 40 cycles of 92 °C for 15 s and 60 °C for 1 min. Relative gene expression levels were calculated using the 2^−ΔΔCt^ method. All experiments were performed in triplicate.

### 4.4. Western Blot

Cells were harvested and lysed using ice-cold lysis buffer containing Tris-HCl, SDS, β-mercaptoethanol, and glycerol. Protein concentration was measured using a BCA Protein Assay Kit (Pierce, Rockford, IL, USA, Cat No. 23225). Equal protein amounts were separated by SDS-PAGE and transferred to PVDF membranes. The membranes were blocked with 5% skim milk powder in TBS-T for 1 h and then incubated overnight at 4 °C with primary antibodies: *KIF18B* (Abcam, Cambridge, UK, Cat No. ab168812, 1:1000), GAPDH (Proteintech, Rosemont, IL, USA, Cat No. 60004-1-Ig, 1:30,000), Skp2 (Proteintech, Rosemont, IL, USA, Cat No. 15010-1-AP, 1:2000), JPH1 (Abcam, Cambridge, UK, Cat No. ab195312, 1:1000), CNN2 (Proteintech, Rosemont, IL, USA, Cat No. 21073-1-AP, 1:1500), EZR (Invitrogen, Carlsbad, CA, USA, Cat No. 35-7300, 1:3000), KIF2C (Proteintech, Rosemont, IL, USA, Cat No. 28372-1-AP, 1:1000), CHSY1 (Proteintech, Rosemont, IL, USA, Cat No. 14420-1-AP, 1:500), and FAM111B (NOVUS, Centennial, CO, USA, Cat No. NBP1-86645, 1:1000). After washing with TBS-T, the membranes were incubated with goat anti-rabbit secondary antibody (Beyotime, Shanghai, China, Cat No. A0208, 1:3000) at room temperature for 2 h. Protein bands were detected using enhanced chemiluminescence (Thermo Fisher Scientific Inc., Waltham, MA, USA). All experiments were performed in triplicate.

### 4.5. Cell Migration and Invasion Assays

Cell migration and invasion were assessed using 6.5 mm Transwell inserts (Corning, NY, USA, Cat. #3422). Equal numbers of cells were seeded into each Transwell insert to ensure comparable initial cell density across all experimental groups. Cells (5 × 10^4^) in 100 μL of serum-free medium were seeded into the upper chamber, while the lower chamber contained complete medium supplemented with 10% serum. After 48 h of incubation at 37 °C in a 5% CO_2_ atmosphere, the cells that did not migrate and remained on the upper surface were gently removed. The membranes were then fixed with 4% paraformaldehyde and stained with 0.1% crystal violet. Migratory cells in four random fields were counted under a microscope. Each experiment was performed in triplicate. For invasion assays, the Transwells were pre-coated with Matrigel (BD Biosciences, San Jose, CA, USA, Cat. #356234) and cells were allowed to invade for 16 h. All experiments were performed in triplicate.

### 4.6. Wound-Healing Assay

HOS and U-2OS cells (5 × 10^4^) were transfected and seeded in 96-well plates. Once the cells reached approximately 90% confluence, a 10 μL pipette tip was used to create a wound in the cell monolayer. The cells were then washed three times with 1× PBS and cultured in serum-free medium for 48 h. Images of wound closure were taken at 48 h using an Olympus microscope(Olympus, Tokyo, Japan). All experiments were performed in triplicate.

### 4.7. The CCK-8 Assay

Cell proliferation was evaluated using a Cell Counting Kit-8 (CCK-8) assay (Sigma-Aldrich, St. Louis, MO, USA). HOS and U-2OS cells with stable vector transfection were digested and seeded into 96-well plates at a density of 5 × 10^3^ cells per well in 200 μL complete culture medium. At indicated time points (days 1–5), 10 μL CCK-8 reagent (5 mg/mL; Sigma-Aldrich, St. Louis, MO, USA) was added to each well, followed by incubation at 37 °C for 4 h. Cell viability was determined by measuring absorbance at 450 nm using a microplate reader. All experiments were performed in triplicate.

### 4.8. Flow Cytometry Analysis

Flow cytometry was conducted to assess cell cycle progression and apoptosis.For cell cycle analysis, cells were collected and fixed in 70% ethanol at 20 °C for 24 h. After fixation, propidium iodide staining solution was added to achieve a final concentration of 100 μg/mL. Samples were incubated in the dark for 10 min and subsequently washed with PBS containing 0.5% BSA prior to analysis using a FACSCalibur flow cytometer (BD Biosciences, Franklin Lakes, NJ, USA). Apoptosis was evaluated using an Annexin V-FITC apoptosis detection kit (BD Biosciences) according to the manufacturer’s protocol. Cells were washed, resuspended in PBS, and incubated with staining solution at room temperature for 20 min in the dark. Samples were immediately analyzed by flow cytometry using a FACScan system (BD Biosciences, San Jose, CA, USA). All experiments were performed in triplicate.

### 4.9. Co-Immunoprecipitation

Co-immunoprecipitation assays were carried out at 4 °C throughout the procedure. MNNG/HOS and U-2OS cells were rinsed twice with pre-chilled PBS and lysed in ice-cold lysis buffer for 5–10 min. Lysates were centrifuged at 13,000× *g* for 10 min to obtain total protein, and protein concentrations were determined using a BCA Protein Assay Kit. Equal amounts of protein lysate were transferred into microcentrifuge tubes, followed by incubation with either 1 μg rabbit IgG (control) or 1 μg specific immunoprecipitating antibody overnight. Subsequently, 20 μL Protein A/G PLUS-Agarose beads were added to each sample and incubated for 1–2 h to capture immune complexes. Normal IgG was used as a negative control to assess nonspecific binding. All experiments were performed in triplicate.

### 4.10. In Vivo Experiments

Twenty-four NSG mice (aged 4–6 weeks) were purchased from Jiangsu GemPharmatech Co., Ltd. (Nanjing, China; License No. SCXK (Su) 2023-0009). (SCXK (Su) 2023-0009) were subcutaneously injected in the flank with HOS cells (1 × 10^7^/200 μL) transfected with *KIF18B*, *KIF18B* + sh*SKP2*, NC, and sh*SKP2*. Tumor volumes were measured using calipers, and tumors were collected for immunohistochemical (IHC) examination and Western blotting.

### 4.11. Clinical Sample Collection and Histological Evaluation Title

#### 4.11.1. Clinical Samples

Formalin-fixed paraffin-embedded (FFPE) specimens were available from 74 patients, who were diagnosed with OS through surgical resection and pathological examination; patients were recruited from the Fourth Affiliated Hospital of Harbin Medical University. Sixty-six surgical specimens from these patients comprised the OS group, while specimens of adjacent normal bone tissue from eight of these patients were designated as the bone tissue group.

#### 4.11.2. Histology Analysis

After obtaining OS tissue, it was rinsed with PBS to remove excess debris. The tissue was then fixed in 4% paraformaldehyde, followed by dehydration through a graded ethanol series. Once dehydrated, the tissue was embedded in paraffin. Paraffin-embedded tissue blocks were sectioned using a microtome and the resulting slices were prepared for subsequent staining. All experiments were performed in triplicate.

### 4.12. Genome-Wide Gene Expression Profiling

#### 4.12.1. Differential Expression Analysis

Differentially expressed genes (DEGs) were identified using the limma package (version 3.50.0) in R (version 4.2.1). Genes with |fold change (FC)| ≥ 1.3 and false discovery rate (FDR) < 0.05 (Benjamini–Hochberg correction) were considered statistically significant.

#### 4.12.2. Pathway and Functional Enrichment Analysis

Ingenuity Pathway Analysis (IPA; QIAGEN, Redwood City, CA, USA; version 01-21-04) was utilized to explore canonical pathways and disease/function annotations. DEGs (gene symbols, log_2_ fold change, and FDR-adjusted *p*-values) were uploaded for IPA. Canonical pathways with *p*-value < 0.05 (Fisher’s exact test) and |Z-score| ≥ 2 were considered significantly activated or inhibited. For disease and functional enrichment, terms with a *p*-value < 0.01 were selected.

### 4.13. Glycolysis Experiment

#### 4.13.1. Intracellular ATP Level Measurement

ATP levels were quantified using the CellTiter-Glo Luminescent Cell Viability Assay (Promega, Madison, WI, USA; G7570) following the manufacturer’s protocols. Briefly, cells were seeded in 96-well white plates (5 × 10^3^ cells/well) and treated as indicated. After 24 h, 100 μL of CellTiter-Glo reagent was added to each well, mixed for 2 min, and incubated for 10 min at room temperature. Luminescence was measured using a SpectraMax M5 microplate reader (Molecular Devices, San Jose, CA, USA). ATP concentrations were normalized to total protein content (BCA assay, Pierce). All experiments were performed in triplicate.

#### 4.13.2. Glucose Uptake Assay

Glucose consumption was determined using the Glucose Assay Kit (Abcam, ab65333) based on the glucose oxidase method: Cells (1 × 10^6^) were cultured in 96-well plates for 24 h. Culture medium was replaced with fresh DMEM containing 2% FBS. After 12 h, the medium was collected and centrifuged (1000× *g*, 5 min) to remove cell debris. Glucose levels in the medium were measured by incubating 50 μL of the sample with 50 μL reaction mix (containing glucose oxidase and peroxidase) at 37 °C for 30 min. Absorbance at 505 nm was recorded, and glucose concentration was calculated against a standard curve. The results were normalized to the cell number, which was counted using a hemocytometer (Marienfeld, Lauda-Königshofen, Germany). All experiments were performed in triplicate.

#### 4.13.3. Lactate Production Assay

Lactate accumulation in the culture medium was quantified using the L-Lactate Assay Kit (Sigma-Aldrich, MAK064): Cell culture supernatants were collected after 24 h of incubation and filtered through 0.22 μm membranes. Then, 10 μL of the sample was mixed with 90 μL of reaction buffer (containing lactate dehydrogenase and NAD^+^). The mixture was incubated at 37 °C for 30 min, and absorbance at 570 nm was measured. Lactate concentration was determined via a standard curve (0–10 mM) and normalized to total protein content. All experiments were performed in triplicate.

### 4.14. Statistical Analyses

Data were analyzed using GraphPad Prism 8.03. All experiments were conducted in triplicate, and results are presented as the mean ± standard deviation (SD). One-way ANOVA was applied for comparisons among multiple groups, followed by Tukey’s post hoc test for pairwise comparisons. A *p*-value of less than 0.05 was considered statistically significant.

## 5. Conclusions

*KIF18B* is overexpressed in OS cells and tissues, and its expression positively correlates with the malignant potential of HOS and U-2OS cells. In clinical tissue samples from patients, *KIF18B* expression shows a positive correlation with N-stage and clinical staging. *KIF18B* promotes OS cell proliferation in vitro. We initially identified an interaction between *KIF18B* and Skp2 through co-immunoprecipitation (Co-IP) assays. These findings suggest that *KIF18B* may be involved in the stabilization of Skp2, as *KIF18B* knockdown accelerated Skp2 ubiquitination and degradation. Functionally, Skp2 is indispensable for *KIF18B*-mediated tumorigenesis, as demonstrated by phenotypic rescue experiments. Knockdown of either *KIF18B* or *SKP2* significantly suppresses OS cell viability and glycolytic metabolism. The ability of *KIF18B* to enhance glycolysis is largely dependent on Skp2. Metabolic reprogramming driven by the *KIF18B*-Skp2 axis facilitates the progression of OS cells.

## Figures and Tables

**Table 1:** Expression patterns in OS tissues and normal tissues revealed by immunohistochemical analysis.

*KIF18B* Expression	Tumor Tissue		Normal Tissue		*p*-Value
	Cases	Percentage	Cases	Percentage	
Low	31	47.0%	8	100%	0.000 ***
High	35	53.0%	0	-	

The data were expressed as mean ± SD (n ≥ 3). *** *p* < 0.001.

**Table 2:** Association between *KIF18B* expression and clinicopathological characteristics in osteosarcoma patients (Mann–Whitney U test).

Features	No. of Patients	*KIF18B* Expression		*p*-Value
		Low	High	
All patients	66	31	35	
Age (years)				0.635
<28	32	16	16	
≥28	34	15	19	
Gender				0.919
Male	43	20	23	
Female	23	11	12	
Tumor size				0.545
≤6 cm	30	10	20	
>6 cm	14	6	8	
T Infiltrate				0.456
T1	30	11	19	
T2	31	20	11	
T3	5	0	5	
lymphatic metastasis (N)				0.016 *
N0	60	31	29	
N1	6	0	6	
Stage				0.006 **
1	2	2	0	
2	58	29	29	
4	6	0	6	

The data were expressed as mean ± SD (n ≥ 3). * *p* < 0.05 and ** *p* < 0.01.

**Table 3:** Correlation between *KIF18B* expression and clinicopathological characteristics in OS patients (Spearman’s rank correlation analysis).

		*KIF18B*
Lymphatic metastasis (N)	Spearman’s rank-order correlation	0.298
	Two-tailed significance level	0.015 *
	N	66
Stage	Spearman’s rank-order correlation	0.343
	Two-tailed significance level	0.005 **
	N	66

The data were expressed as mean ± SD (n ≥ 3). * *p* < 0.05 and ** *p* < 0.01.

## Data Availability

Data supporting the present study are available from the corresponding author upon reasonable request.

## Abbreviations

The following abbreviations are used in this manuscript:

AJCC	American Joint Committee on Cancer
CHX	cycloheximide
Co-IP	co-immunoprecipitation
DEGs	differentially expressed genes
FBXL1	F-box and leucine-rich repeat protein 1
FFPE	formalin-fixed paraffin-embedded
GLUT	glucose transporter
HE	hematoxylin–eosin
IHC	immunohistochemical
IPA	Ingenuity Pathway Analysis
*KIF18B*	kinesin family member 18B
MCAK	Mitotic Centromere-Associated Kinesin
OS	osteosarcoma
Skp2	S-phase kinase-associated protein 2
TCA	tricarboxylic acid cycle
Ub	ubiquitin
UPS	ubiquitin–proteasome system
